# Resting state in Parkinson's disease dementia and dementia with Lewy bodies: commonalities and differences

**DOI:** 10.1002/gps.4342

**Published:** 2015-08-13

**Authors:** Luis R. Peraza, Sean J. Colloby, Michael J. Firbank, G. Shirmin Greasy, Ian G. McKeith, Marcus Kaiser, John O'Brien, John‐Paul Taylor

**Affiliations:** ^1^ Institute of Neuroscience, Campus for Ageing and VitalityNewcastle UniversityNewcastle upon TyneUK; ^2^ Institute of Ageing, Campus for Ageing and VitalityNewcastle UniversityNewcastle upon TyneUK; ^3^ Interdisciplinary Computing and Complex BioSystems (ICOS) Research Group, School of Computing ScienceNewcastle UniversityNewcastle upon TyneUK; ^4^ Department of Psychiatry, Cambridge Biomedical CampusUniversity of Cambridge School of Clinical MedicineCambridgeUK

**Keywords:** default mode, cognitive fluctuations, attention, networks, fMRI

## Abstract

**Objective:**

Dementia with Lewy bodies (DLB) and Parkinson's disease dementia (PDD) are two dementias with overlapping phenotypes. Clinically, these are differentiated by the one‐year precedence rule between the onset of dementia with respect to Parkinsonism. In this report we aimed to find differences between DLB and PDD in functional connectivity (FC) using resting state functional magnetic resonance imaging, which we hypothesised would reflect the underlying pathological differences between DLB and PDD.

**Methods:**

The study cohort comprised of 18 patients with DLB, 12 with PDD and 17 healthy control (HC) groups. Eight cortical and four subcortical seeds were chosen, and time series extracted to estimate correlation maps. We also implemented a voxel‐based morphometry (VBM) analysis to assess regional grey matter differences. FC analysis was corrected for age, sex and regional grey matter differences.

**Results:**

The FC analysis showed greater alterations in DLB than in PDD for seeds placed within the fronto‐parietal network (FPN), whilst in contrast, for the supplementary motor area seed FC alterations were more apparent in PDD than in DLB. However, when comparing DLB and PDD, no significant differences were found. In addition, VBM analysis revealed greater atrophy in PDD than HC and DLB in the bilateral motor cortices and precuneus respectively.

**Conclusions:**

PDD and DLB demonstrate similar FC alterations in the brain. However, attention‐ and motor‐related seeds revealed subtle differences between both conditions when compared with HC, which may relate to the neuropathology and chronological precedence of core symptoms in the Lewy body dementias. © 2015 The Authors *International Journal of Geriatric Psychiatry* Published by John Wiley & Sons, Ltd.

## Introduction

Lewy body dementias are considered to be the second to third most common cause of dementia after Alzheimer's disease (AD), representing 10–15% of all late onset dementia cases (Ballard *et al*., 2011). This umbrella term is applied to define two symptomatically overlapping types of dementias, dementia with Lewy bodies (DLB) and Parkinson's disease dementia (PDD). Although both dementias belong to the same Lewy body spectrum (McKeith, 2007) showing similar neuropathological basis (Spillantini and Goedert, 2000; Tsuboi *et al*., 2007) and common symptoms (e.g. cognitive/attentional fluctuations, complex visual hallucinations or VHs and Parkinsonism), previous neuroimaging research has reported functional and structural differences between the two diseases. Currently, it is known that DLB presents with higher amyloid burden than PDD (Brooks, 2009; Gomperts, 2014), which is believed to be responsible of the more AD‐like symptoms and the rapid progression of dementia (Edison *et al*., 2008). Furthermore, structural imaging has found that patients with DLB have greater brain tissue atrophy compared with PDD (Beyer *et al*., 2007; Lee *et al*., 2010b; Lee *et al*., 2010a).

From a neuropsychological perspective patients with DLB tend to have greater impairments in attention and executive function (Gnanalingham *et al*., 1997; Downes *et al*., 1999) as well as visual recognition memory than patients with PDD (Mondon *et al*., 2007). In addition, more severe Parkinsonism is associated with PDD compared with DLB. These clinical and neuroimaging investigations suggest that despite a common aetiology, there may be pathological differences between both conditions.

One approach for investigating differences between DLB and PDD is resting state functional magnetic resonance imaging (rs‐fMRI). Analysis of rs‐fMRI searches for spatial correlations in the blood oxygenation level‐dependent signal, which are associated with neural synchronizations (Lobotesis *et al*., 2001) and thus can provide measures of functional brain activity. Specifically, we were interested in the rs‐fMRI connectivity from three resting state networks associated with reported clinical differences in attention/executive function, memory and motor control between PDD and DLB; the default mode network (DMN), the fronto‐parietal network (FPN) and the motor network (MN). The DMN is involved in recollection of autobiographical events and mind wandering (Raichle *et al*., 2001; Mevel *et al*., 2011), and previous studies in AD have demonstrated that disruptions of this network are related to memory deficits (Wang *et al*., 2007; Binnewijzend *et al*., 2012). The FPN is highly engaged in attention (Fox *et al*., 2006) and executive control (Heine *et al*., 2012) and has been suggested to be targeted by Lewy body pathology (Franciotti *et al*., 2013). Finally, disruptions in the MN (Robinson *et al*., 2009) have been associated with Parkinson's disease (PD), with alterations in basal ganglia and thalamo‐cortical loops observed in these patients (Kwak *et al*., 2010; Baudrexel *et al*., 2011; Hacker *et al*., 2012).

Such rs‐fMRI networks or related regions have been previously investigated in Lewy body dementias where functional connectivity (FC) alterations in PDD (Rektorova *et al*., 2012; Seibert *et al*., 2012; Tessitore *et al*., 2012; Baggio *et al*., 2015) and DLB (Galvin *et al*., 2011; Kenny *et al*., 2012; Kenny *et al*., 2013; Lowther *et al*., 2014; Peraza *et al*., 2014) have been reported when comparing against healthy control (HC) groups. However, to date there have been no studies assessing rs‐fMRI differences between PDD and DLB.

In the present report, we therefore used rs‐fMRI to study FC between patients with DLB and PDD using a seed‐based approach in regions related to the FPN, DMN and MN, and for comparison, we included a HC group. We hypothesised that rs‐fMRI would be able to detect altered FC patterns in DLB and PDD compared with HCs as well as between dementia groups.

## Methods

### Subjects and assessment

The study comprised 55 participants: 22 diagnosed with DLB, 16 diagnosed with PDD and 17 HCs. Diagnosis of patients was assessed independently by two experienced clinicians according to consensus diagnostic criteria for DLB and PDD (McKeith *et al*., 2005; Emre *et al*., 2007). From the DLB group, nine patients had dopaminergic imaging and all of them showed abnormal uptake. Clinical assessment included the Mini‐Mental State Examination (MMSE), Cambridge Cognitive Examination (CAMCOG), Neuropsychiatric Inventory (NPI) (Cummings *et al*., 1994), Unified Parkinson's Disease Rating Scale (UPDRS) and the Clinical Assessment of Fluctuations (CAF) (Walker *et al*., 2000). Furthermore, in order to assess the level of complex VHs, the hallucination‐subscale questionnaire of the NPI (NPI_hall_) was completed by the patient carers. The control group had no history of psychiatric or neurological disorders (MMSE > 27). Approval for this study was granted by the Newcastle Ethics Committee, and all participants gave informed consent.

### MRI and fMRI acquisition

Brain images were recorded using a 3 T Philips Intera Achieva MRI scanner. Structural images were obtained with a magnetisation prepared rapid gradient‐echo sequence, sagittal acquisition, echo time 4.6 ms, repetition time 8.3 ms, inversion time 1250 ms, flip angle = 8°, sensitivity encoding factor = 2 and in‐plane field of view 240 × 240 mm with slice thickness 1.0 mm (voxel of 1.0 × 1.0 × 1.0 mm). For the rs‐fMRI, participants laid within the MRI scanner with eyes open and images were obtained with a gradient‐echo echo‐planar imaging sequence with 25 contiguous axial slices, 128 volumes, in‐plane resolution = 2 × 2 mm, slice thickness = 6 mm, repetition time = 3000 ms and field of view = 260 × 260 mm.

### fMRI pre‐processing and seed extraction

Participants' fMRIs were first pre‐processed using the FMRIB Software Library (FSL 5.0; www.fmrib.ox.ac.uk/fsl). This included FMRIB's Linear Image Registration Tool for motion correction with spatial smoothing full width at half maximum of 6.0 mm and high‐pass filter of 150 s. Then, motion parameters were analysed for exclusion criteria: translation >2 mm and rotation >1° (Liao *et al*., 2010; Ni *et al*., 2012), and comparisons between groups for motion and rotation were evaluated by the motion/rotation formula (Liao *et al*., 2010); motion/rotation = ${\left(M-1\right)}^{-1}\sum_{i=2}^{M}\sqrt{\left|x_{i}\right.-x_{i-1}\left|{}^{2}\right.+\left|y_{i}\right.-y_{i-1}\left|{}^{2}\right.+\left|z_{i}\right.-z_{i-1}\left|{}^{2}\right.}$, where *x*, *y* and *z* are the parameters for either translations or rotations and *M* is the fMRI length. Artefact denoising was implemented using independent component analysis (Multivariate Exploratory Linear Optimized Decomposition into Independent Components) with standardised criteria (Kelly *et al*., 2010); artefacts that resembled movements, cerebro‐spinal fluid or whose power spectra were widespread through all frequencies or above 0.10 Hz were filtered out.

Then, structural and functional images were coregistered and normalised to MNI (Montreal Neurological Institute) space using Statistical Parametric Mapping (SPM8, http://www.fil.ion.ucl.ac.uk/spm/) and fMRI images were resampled to 2 × 2 × 2 mm voxels.

A total of 12 seeds were chosen for our study because of their relation to the DMN, FPN and MN. For the DMN we chose the middle posterior cingulate cortex (mPCC, MNI 0,−51,29), medial prefrontal cortex (mPFC, MNI 0,61,22) and the medial precuneus cortex (mPrC, MNI 0,−58,48) (Damoiseaux *et al*., 2008). For the FPN, seeds were placed at the posterior aspect of the FPN, which covers the parietal cortices. The FPN is the only lateralised resting state network, but its posterior aspect is present bilaterally (Fox *et al*., 2006). FPN seeds were left/right posterior intraparietal sulcus (lpIPS, MNI −26, −65,52; rpIPS, MNI 28,−65,52) and left/right anterior intraparietal sulcus (laIPS, MNI −45, −37,46; raIPS, MNI 43,−36,46) (Brier *et al*., 2012; Markett *et al*., 2014). For the MN seeds, we chose bilateral putamen (left/right Put, MNI ±26,−2,9), thamalus (left/right Thal, MNI ±12,−18,7) and supplementary motor area (SMA, MNI 1,−6,55).

With the seed definitions, times series extraction and *z*‐score images were obtained using REST software, version 1.8 (Song *et al*., 2011). fMRIs were detrended and low‐pass filtered (0.10 Hz) before time series extraction. Cortical and subcortical seeds were created with 6‐ and 4‐mm radius spheres respectively.

### Voxel‐based morphometry

In order to assess whether grey matter atrophy may confound the FC results in our study, we ran a voxel‐based morphometry (VBM) analysis using SPM8 implementing the DARTEL registration algorithm (Ashburner, 2007). DARTEL maps were then smoothed with an 8‐mm full width at half maximum spatial filter. For all participants, estimates of total intracranial volume were also calculated and used as covariates in the VBM analysis, and DARTEL maps were spatially down sampled to enable their use as voxel‐wise covariates in the FC analysis.

### Statistical analysis

Analysis of demographic and clinical variables was carried out using SPSS (version 21 SPSS, IBM). Age at onset of Parkinsonism minus age at onset of cognitive symptoms (PD‐CI) was assessed for differences between PDD and DLB with a Mann–Whitney test. Statistical comparisons of the motion parameters (translations and rotations) for the three groups were assessed by Kruskal–Wallis tests.

Grey matter volume differences were assessed first by an ANCOVA for the three groups, followed by post hoc unpaired two‐sample *t*‐tests. For all VBM analyses, age, sex and total intracranial volume were included as covariates (Watson *et al*., 2012).

Between group comparisons for FC were assessed by two‐sample unpaired *t*‐tests with non‐parametric permutations (FSL‐randomise, 5000 permutations), correcting for age, sex and regional grey matter. The latter was included as a voxel‐wise covariate. All results were considered significant at *p*‐value < 0.05 corrected for multiple comparisons using threshold free cluster enhancement (TFCE).

## Results

From the 55 participants in our study, four patients with DLB and four patients with PDD were excluded because of excessive motion (>2 mm translation or >1° rotation), leaving a remaining cohort of 18 DLBs, 12 PDDs and 17 HCs. Statistical analysis of the motion parameters for the remaining groups revealed no significant differences (Kruskal–Wallis test: motion *p*‐value = 0.142, *χ* ^2^ = 3.91, df = 2; rotation *p*‐value = 0.56, *χ* ^2^ = 1.14, df = 2).

### Demographic and clinical data

Demographic and clinical data are shown in Table 1. The PDD group was younger than both DLB and HC groups (*p*‐value = 0.022, ANOVA test). However, both dementia groups were cognitively matched (mild to moderate impairment), had similar levels of frequency/severity of complex VHs as reported by the NPI_hall_ score and were also matched for cognitive fluctuations frequency/severity, although there was a trend for higher CAF scores in the PDD group (*p*‐value = 0.08). Executive and attention CAMCOG subscores were compared between dementia groups with no significant differences. As expected, the PDD group showed greater Parkinsonism (*p*‐value < 0.007) compared with DLB, and PD‐CI was significantly different between PDD and DLB (*p*‐value < 0.001); cognitive impairment in DLB occurred on average 0.53 years after onset of Parkinsonism, whilst in PDD, onset of Parkinsonism occurred on average 7.5 years before onset of cognitive impairment. Both patient groups matched by the duration in years of cognitive symptoms (*p*‐value = 0.366).

**Table 1:** Demographic and clinical data for the three groups

	HC (*n* = 17)	DLB (*n* = 18)	PDD (*n* = 12)	*p*‐value
Gender M:F	14:3	13:5	11:1	*χ* ^2^ = 1.79, *p* = 0.407^a^
Age	76.88 ± 5.8	77.17 ± 6.1	71.5 ± 4.8	*F*(2,44) = 4.2, *p* = 0.022^b^
MMSE	29.1 ± 0.9	23.6 ± 3.9	22.5 ± 5.2	*t*_28_ = 0.66, *p* = 0.51^c^
CAMCOG	96.4 ± 3.3	76.2 ± 13	75.8 ± 14.6	*t*_28_ = 0.07, *p* = 0.94^c^
CAMCOG atten	6.7 ± 0.56	4.5 ± 2.12	4.0 ± 2.0	*t*_28_ = 0.64, *p* = 0.52^c^
CAMCOG exec	22.9 ± 2.7	13.5 ± 5.12	14.17 ± 1.94	*t*_28_ = 0.42,*p* = 0.67^c^
NPI hall	na	1.65 ± 1.83^d^	2.6 ± 2.3	*t*_27_ = 1.21, *p* = 0.23^c^
UPDRS	1.41 ± 1.87	17.4 ± 7.8	26.4 ± 9.0	*t*_28_ = 2.9, *p* < 0.007^c^
CAF	na	3.29 ± 4.0^d^	6.1 ± 3.4^e^	*t*_25_ = 1.83, *p* = 0.08^c^
PD‐CI years	na	0.53 ± 1.8^f^	−7.5 ± 4.42	*p* < 0.001^g^
Cog. symptoms years	na	3.63 ± 2.58	2.79 ± 1.7	*p* = 0.366^g^

Values expressed as mean ± 1 SD.

Abbreviations: HC, healthy controls; DLB, dementia with Lewy bodies; PDD, Parkinson's disease dementia; MMSE, Mini‐Mental State Examination; CAMCOG, Cambridge Cognitive Examination; CAMCOG atten, attention subscore; CAMCOG exec, executive subscore; NPI, Neuropsychiatric Inventory; CAF, Clinical Assessment of Fluctuations; UPDRS, Unified Parkinson's Disease Rating Scale; PD‐CI years, age at onset of Parkinsonism minus age at onset of cognitive impairment; cog‐symptoms, duration of cognitive impairment in years; na, not applicable.

a Chi‐square test.

b Anova.

c Student's *t*‐test – DLB vs PDD.

d *n* = 17.

e *n* = 10.

f *n* = 15.

g Mann–Whitney *U*‐test.

### Regional grey matter volume differences

**Figure 1:**
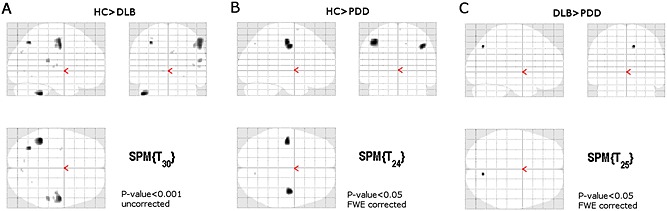
Voxel‐based morphometry analysis in DLB and PDD. (A) The DLB group showed grey matter volume loss at the left motor cortex, parietal and cerebellar regions (at *p*‐uncorrected < 0.001) when compared against the healthy control (HC) group. (B) The PDD group showed significant grey matter loss in bilateral motor cortices compared with healthy controls (at *p*‐value < 0.05 corrected). (C) Differences between the DLB and PDD groups were found the right precuneus cortex only (MNI 10, −70, 42). Glass brains are presented in neurological convention (left is left hemisphere and right is right hemisphere).

Results demonstrated a spatially small but significant difference in the right precuneus cortex (MNI 10,−70,42, *p*‐value < 0.05 corrected, ANCOVA). Subsequently, we ran between group comparisons whose results are shown in Figure 1. DLB > PDD and HC > PDD were the only tests that survived correction for multiple comparisons (*p*‐value < 0.05 corrected, Figures 1(b) and 1(c)), whilst for HC > DLB regions were significantly different but only at the uncorrected level (*p*‐value < 0.001; Figure 1(a)). For DLB > HC, PDD > HC and PDD > DLB, results were not significant (*p*‐uncorrected value < 0.001).

### Functional alterations in DLB

**Figure 2:**
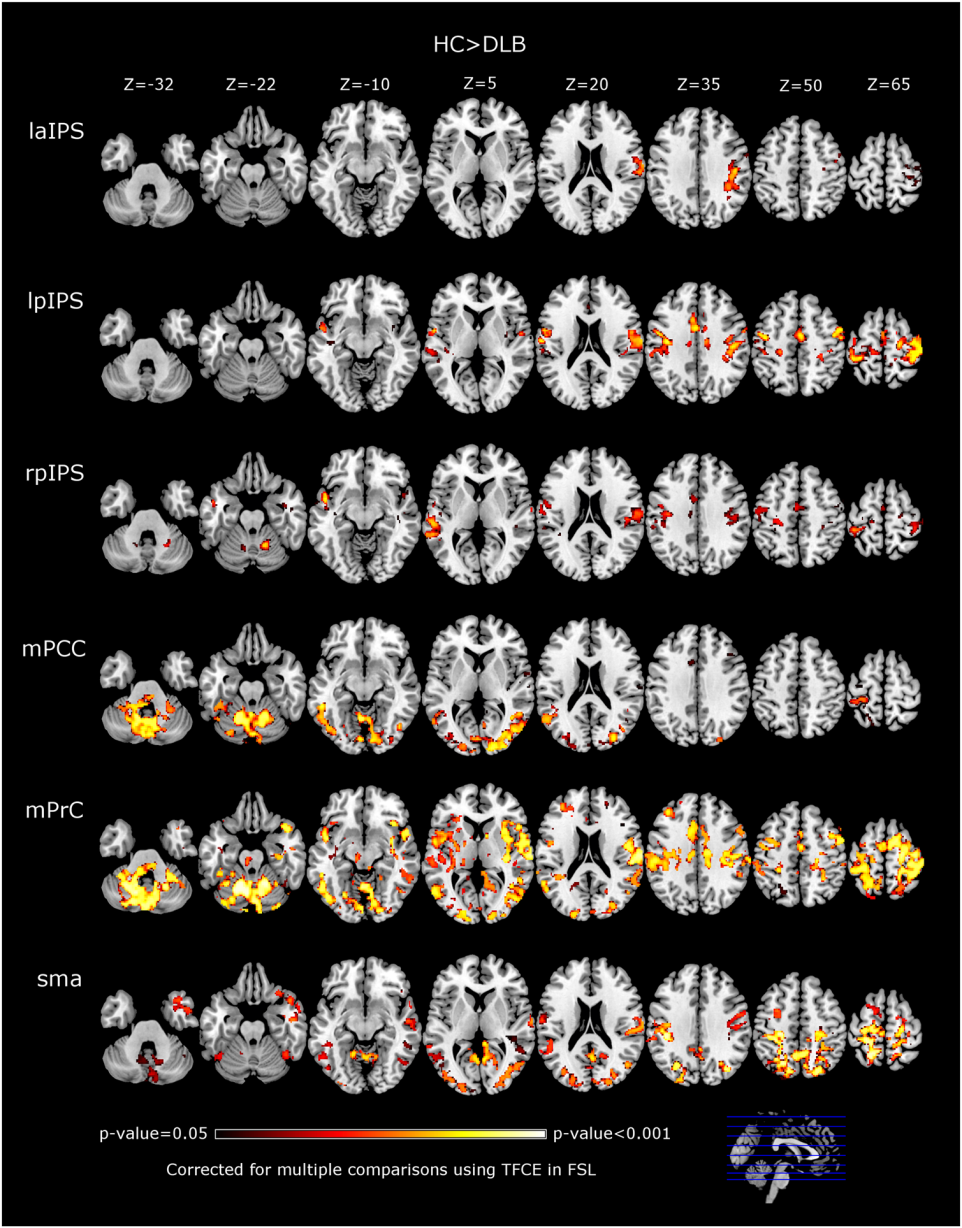
Functional connectivity (FC) alterations in DLB compared with healthy controls (HC). For analysed seeds the DLB showed lower FC than healthy controls (at *p*‐value < 0.05, TFCE corrected for multiple comparisons). Brains presented in MNI axial views in neurological convention. Seeds: l/rpIPS: left and right posterior intraparietal sulcus, laIPS: left anterior intraparietal sulcus, mPCC: medial posterior cingulate cortex, mPrC: medial precuneus cortex, SMA: supplementary motor area.

FC results for the DLB group are shown in Table 2 and Figure 2. DMN‐related seeds (mPCC, mPFC and mPrC) revealed disconnections with cerebellar regions where the highest significant difference was found. Other significant regions were intracalcarine cortices, lingual gyri, lateral occipital cortices, temporal cortices and cingulate gyri. For the mPrC seed, significant lower FC (HC > DLB) was also seen in the thalamus and pallidum.

**Table 2:** Significant clusters and regions for HC > DLB comparisons^a^

Seed	*p*‐value	MNI	Voxels	Brain regions
laIPS	0.014	46,−20,36	1161	R postcentral gyrus*
				R supramarginal gyrus posterior division
				R superior parital lobule
	0.04	28,−26,70	189	R precentral gyrus*
	0.041	30,−16,68	78	R precentral gyrus*
	0.045	−48,−4,36	11	L precentral gyrus*
lpIPS	0.01	−38,−14,34	8545	L precentral gyrus*, R precentral gyrus, L/R postcentral gyrus, L/R superior temporal gyrus, L/R central opercular cortex, L/R supplementary motor cortex, L/R cingulate cortex, anterior division
	0.037	38,0,−2	141	R Insular cortex
	0.042	0,30,22	59	L cingulate gyrus*
	0.044	−42,−14,−12	50	L planum polare
	0.043	−56,−14,38	49	L postcentral gyrus*
mPCC	0.007	−12,−64,−30	11 593	Cerebellar left VI*, cerebellum, L/R middle temporal gyrus, L/R angular gyrus, L/R lateral occipital cortex, L/R intracalcarine cortex, brain stem, L/R lingual gyrus
	0.022	−28,−32,66	607	L postcentral gyrus*, L superior parietal lobule
	0.042	56,−14,0	138	R planum temporale*, R central opercular cortex
	0.041	50,20,40	76	R middle frontal gyrus*
	0.04	−10,10,38	59	L paracingulate gyrus*
	0.047	−28, −16, 68	24	L precentral gyrus*
	0.048	64,−20,20	24	R supramarginal gyrus*
	0.049	12,−54,2	15	R Lingual gyrus*
mPFC	0.043	−14,−64,−30	20	Cerebellar left VI*
mPrC	0.001	−14,−64,−30	36 556	Cerebellar left VI*, right VI, cerebellum, brain stem, L/R lingual gyrus, L/R lateral occipital cortex, L/R temporal pole, L/R central opercular cortex, L/R postcentral gyrus, L/R precentral gyrus, L/R thalamus, L/R pallidum
	0.033	22,48,22	119	R Frontal pole*
raIPS	0.04	−50,−2,−14	25	L superior temporal gyrus*
rpIPS	0.01	−50,2,−6	3286	L planum polare*, L planum polare, L superior temporal gyrus, superior division, L precentral gyrus, L postcentral gyrus, L central opercular cortex
	0.022	60,−16,18	637	R central opercular cortex*, R parietal operculum cortex
	0.024	38,−30,60	605	R postcentral gyrus*, R precentral gyrus
	0.02	0,−8,56	568	L supplementary motor cortex*, L cingulate gyrus, anterior division
	0.014	18,−52,−26	273	Cerebellar right V*
	0.041	−36,−40,16	66	L parietal operculum cortex*
	0.035	20,−20,64	53	R precentral gyrus*
	0.036	−14,−56,−18	50	Cerebellar left V*
	0.045	50,−14,−12	44	R superior temporal gyrus, posterior division
	0.039	40,−6,−22	37	R superior temporal gyrus, posterior division
	0.037	48,8,−12	34	R temporal pole*
	0.048	16,−12,40	28	R supplementary motor cortex
	0.046	46,−6,48	27	R precentral gyrus*
	0.048	−36,−16,−8	18	L Insular cortex*
	0.043	−14,−54,−32	18	Cerebellar left VI*
	0.046	12,2,58	16	R supplementary motor cortex
	0.048	22,−32,58	13	R postcentral gyrus*
	0.048	36,−16,66	10	R precentral gyrus*
SMA	0.001	−18,−42,56	17 074	L postcentral gyrus*, R postcentral gyrus, L/R lateral occipital cortex, L/R lingual gyrus, L/R cingulate gyrus posterior division, L/R precuneus cortex
	0.031	10,−66,−36	468	Cerebellar right vIIIa*
	0.038	52,−48,4	296	R middle temporal gyrus*, temporo‐occipital part
	0.041	−4,−84,−38	35	Cerebellar left crus II

a Clusters were considered significant at *p*‐value < 0.05 TFCE corrected for multiple comparisons. Asterisk indicates the regions where the lowest *p*‐value is located.

For FPN‐related seeds, significantly lower FC in DLB compared with controls was mainly found in the motor‐sensory cortices; precentral and postcentral gyri. Other significant regions were the supramarginal gyri, temporal cortices and cerebellum.

Seeds related to the MN did not show significant differences for the subcortical seeds (putamen and thalamus), but the SMA seed revealed lower FC in DLB for the postcentral gyri, lateral occipital cortices, cingulate, precuneal cortices and cerebellum. No regions demonstrated greater connectivity in DLB compared with HCs.

### Functional alterations in PDD

**Figure 3:**
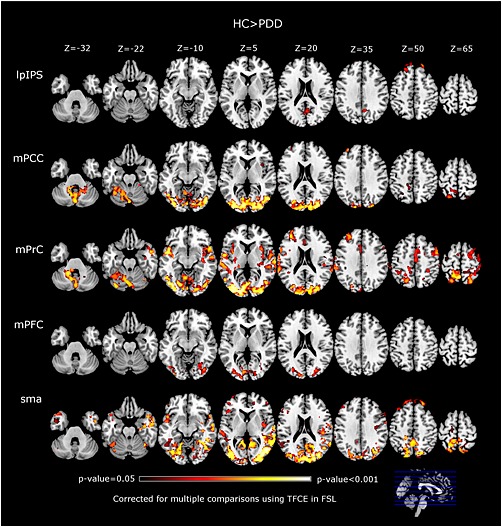
Alterations in functional connectivity (FC) in PDD compared with healthy controls (HCs). The PDD group showed significant lower FC than HCs at several regions for the assessed seeds. Brains presented in MNI axial views in neurological convention. Seeds: lpIPS: left posterior intraparietal sulcus, mPCC: medial posterior cingulate cortex, mPrC: medial precuneus cortex, mPFC: medial prefrontal cortex, SMA: supplementary motor area.

Results for FC alterations in PDD compared with HCs are shown in Table 3 and Figure 3. Seeds related to the DMN revealed lower FC in PDD at occipital regions, specifically the intracalcarine, lateral occipital cortices and lingual gyri. Other significant regions were cerebellum, precuneus, temporal cortices, precentral and postcentral gyri. For the mPCC seed, there was a small cluster showing lower FC with the left thalamus.

**Table 3:** Significant clusters and regions for HC > PDD comparisons^a^

Seed	*p*‐value	MNI	Voxels	Brain regions
lpIPS	0.028	4, −62, 20	436	R precuneus cortex*, cuneal cortex
	0.028	−20, 40, 50	348	L frontal pole*, L superior frontal gyrus
	0.019	−20, 52, 48	82	R frontal pole*,
	0.037	8, 58, 40	64	R frontal pole*
	0.045	−50, 32, 2	30	L inferior frontal gyrus, pars triangularis
	0.045	−34, 62, 6	16	L frontal pole*
mPCC	0.003	−12, −86, 8	11443	L intracalcarine cortex*, R intracalcarine cortex, L/R lateral occipital cortex, inferior and superior divisions, L/R lingual gyrus, cerebellum, vermis VIIIa
	0.02	−18, −66, 68	978	L lateral occipital cortex*, L precuneus cortex, L postcentral gyrus
	0.013	−34, 48, 28	181	L frontal pole*
	0.037	36, 12, 6	93	R insular cortex*
	0.026	−30, −32, −2	84	L thalamus*
	0.043	−54, −24, −2	64	L superior temporal gyrus*
	0.041	22, −68, 64	60	R lateral occipital cortex*
	0.044	38, −4, 0	58	R Insular cortex*
	0.045	32, −54, 56	25	R superior parietal lobule*
	0.047	−64, −36, 20	18	L parietal operculum cortex*
	0.041	52, 4, −18	16	R superior temporal gyrus, anterior division*
mPFC	0.011	40, −74, −6	3789	R lateral occipital cortex, inferior division*, L lateral occipital cortex, L/R lingual gyrus, L/R lateral occipital cortex, superior division
mPrC	0.003	−12, −86, 8	15270	L Intracalcarine cortex*, R intracalcarine cortex, L/R lateral occipital cortex, inferior/superior divisions, L central opercular cortex, L planum polare, L insular cortex, L inferior temporal gyrus, temporooccipital part, cerebellum
	0.008	−4, −56, 66	7319	L precuneus cortex*, L/R superior parietal lobule, L/R supplementary motor area, R precentral gyrus, L/R lateral occipital cortex, superior division.
	0.008	52, 6, −18	3484	R temporal pole*, R superior temporal gyrus, R precentral gyrus
	0.039	−24, −16, 78	171	L precentral gyrus*
	0.047	44, 12, 30	29	R middle frontal gyrus*
	0.049	46, 0, 24	10	R precentral gyrus*
rpIPS	0.042	−42, 48, 8	9	L frontal pole*
	0.047	−22, 52, 40	7	L frontal pole*
SMA	0.001	40, −78, −2	18658	R lateral occipital cortex*, L lateral occipital cortex, L/R lingual gyrus, L/R temporal occipital fusiform cortex, L/R precuneus cortex, R middle temporal gyrus, R superior temporal gyrus, R temporal gyrus, temporooccipital part
	0.017	−48, 14, −10	1766	L temporal pole*, L inferior frontal gyrus, L middle frontal gyrus
	0.019	−66, −22, 4	866	L superior temporal gyrus*, L supramarginal gyrus, posteriordivision
	0.018	24, 44, 50	440	R frontal pole*
	0.036	16, 44, 4	269	R cingulate gyrus*
	0.036	58, 20, 38	147	R precentral gyrus*
	0.046	−6, 64, 40	129	L frontal pole*
	0.036	−48, 2, −32	129	L temporal pole*
	0.029	−36, 62, 2	103	L frontal pole*
	0.042	−14, −22, 66	87	L precentral gyrus*
	0.024	58, 28, 26	70	R middle frontal gyrus*
	0.034	48, 48, 6	51	R frontal pole*
	0.045	−20, 54, 0	33	L frontal pole*
	0.039	−4, 68, 0	30	L frontal pole*
	0.032	−64, −6, 24	26	L postcentral gyrus*
	0.042	32, −52, 74	21	R superior parietal lobule*
	0.048	−48, −50, 28	14	L angular gyrus
	0.044	−50, −4, 56	10	L precentral gyrus*

a Clusters were considered significant at *p*‐value < 0.05 TFCE corrected for multiple comparisons. Asterisk indicates the region where the lowest *p*‐value is located.

Seeds related to the FPN revealed much less differences in PDD than in DLB when compared with HCs. Only the lpIPS and rpIPS showed significant clusters covering few regions: frontal poles, precuneus and cuneal cortices.

For seeds related to the MN, results were similar to the DLB group. Subcortical seeds did not reveal differences with the HC group, and only the SMA seed showed broader regions with significantly lower FC for PDD than DLB when compared with HCs. Significant regions were primarily located in the occipital lobes, specifically the lateral occipital cortices and lingual gyri (Table 3).

As in the DLB group, PDD did not exhibit higher FC than the HC group for any of the seeds assessed.

### Functional differences between DLB and PDD

Comparisons between patient groups did not reveal any significant FC differences (PDD > DLB and DLB > PDD (*p*‐value < 0.05 corrected).

## Discussion

We report significant alterations in FC in DLB and PDD compared with HCs in rs‐fMRI for seeds located within known resting state networks relevant to clinical differences between DLB and PDD; DMN, FPN and MN. Our results show that both patient groups have lower FC than HCs and, while there were differences in the patterns of FC disconnectivity between DLB and PDD compared with HCs, direct comparison of dementia groups did not yield significant differences.

### Differences in regional grey matter volume

Grey matter loss was observed in motor cortices in PDD compared with HCs, and similar results were reported by Melzer *et al*. (2012). In DLB there was a trend of grey matter loss at the motor, cerebellar and parietal cortices, which agree with previous VBM investigations (Ballmaier *et al*., 2004; Beyer *et al*., 2007; Watson *et al*., 2012).

### Default mode network seeds

Both disease groups showed similar patterns of FC alterations for seeds related to the DMN. The main difference between DLB and PDD was that in DLB the most significant region was located in the cerebellum, whilst in the PDD group the greatest difference was within the occipital cortex.

Significant hypometabolism at posterior cortical regions is well established in Lewy body diseases (Lobotesis *et al*., 2001; Sato *et al*., 2007), and previous studies in rs‐fMRI assessing the DMN have also found similar abnormalities. For example, Rektorova *et al*. (2012) reported lower FC in occipital cortices in PDD when assessing a cuneal seed. Tessitore *et al*. (2012) observed reduced FC in cognitively impaired patients with PD in intraparietal cortices and the right middle temporal lobe when assessing differences in DMN maps against HCs. Yao *et al*. (2014) reported lower FC in patients with PD with VHs in precuneus and frontal poles, and similar findings were reported by Amboni *et al*. (2015) in patients with PD with and without mild cognitive impairment (MCI). In DLB, Lowther *et al*. (2014) reported lower FC when assessing the DMN with the cuneal cortex, lingual gyri and occipital regions. In overview, therefore, our findings appear to largely agree with these previous investigations and point towards a consensus that in Lewy body diseases, resting‐state alterations associated to the DMN tend to occur in posterior brain regions, mainly in occipital, parietal and precuneal cortices.

### Fronto‐parietal network seeds

Results for seeds within the FPN revealed differential results in DLB and PDD compared with HCs. In DLB, there were FC alterations compared with HCs, mainly at precentral and postcentral gyri, temporal, occipital and cerebellar regions, whilst in PDD alterations for these seeds were limited to precuneal and frontal cortices. Few studies in Lewy body diseases have assessed the FPN or seeds related to this network. Baggio *et al*. (2015) assessed the dorsal attentional network (DAN), which is the parietal element of the FPN, and reported lower FC in patients with PD with MCI in frontal, temporal, precentral and postcentral cortices, as well as thalami and left putamen. These alterations were also correlated with attention/executive function scores. Amboni *et al*. (2015) also reported lower FC in bilateral prefrontal cortex for the FPN in patients with PD‐MCI, and Peraza *et al*. (2014) found lower FC in patients with DLB compared with HCs at basal, frontal and occipital regions, which significantly correlated with the composite CAF score (severity times frequency) of cognitive fluctuations in DLB. Furthermore, there is currently a growing trend of research evidence suggesting impaired connectivity between the DMN and FPN and also within the FPN between its ventral and dorsal aspects [DAN and ventral attention network (VAN)]. Certainly, dysfunctional connectivity between DMN, DAN and VAN has been implicated in the aetiology of complex VHs in PD (Shine *et al*., 2014a; Shine *et al*., 2015). In this regard, we also observed reduced FC between the FPN seeds located in the IPS and frontal and precuneal cortices in our participants with PDD, which agree with observations of impaired communication between attention networks and DMN in Lewy body diseases (Shine *et al*., 2014b).

Our results in both PDD and DLB therefore agree with the findings reported in these investigations. However, the broader FC alterations shown in DLB compared with PDD for FPN related seeds may point toward the greater amyloid burden in DLB (Gomperts, 2014) and the network degeneration hypothesis (Seeley *et al*., 2009), which proposes that different neurodegenerative diseases target specific network systems and in this context previous investigations have suggested a predilection in DLB toward FPN dysfunction (Franciotti *et al*., 2013; Peraza *et al*., 2014). It has been speculated that this neuropathological network transmission occurs by transynaptic communications through the brain's structural paths (Masuda‐Suzukake *et al*., 2014), which consequently affects pre‐synaptic transmission and alters FC (Schulz‐Schaeffer, 2010). It is possible therefore that the higher burden of amyloid protein in addition to the presence of pre‐synaptic alpha‐synuclein accelerates FC abnormalities in the FPN in DLB. In PDD the FPN is also altered, see for instance Baggio *et al*. (2014), but perhaps to a lesser extend compared with DLB.

### Motor network seeds

Interestingly, we did not find differences between patient groups and HCs for the thalamic and putaminal seeds. This contrasts with previous studies where FC alterations were found using basal ganglia seeds in Lewy body diseases (Kwak *et al*., 2010; Baudrexel *et al*., 2011; Kenny *et al*., 2013). There may be several factors which explain this variance. First, seed analysis in basal regions may have low sensitivity since the majority of previous findings have been reported as uncorrected for multiple comparisons (Kwak *et al*., 2010; Baudrexel *et al*., 2011; Seibert *et al*., 2012). Second, our patient groups presented with lower Parkinsonism compared with previous investigations, see for instance Hacker *et al*. (2012). Finally, different pre‐processing pipelines and quality controls for motion between groups, such as regression of fMRI global signal, which we did not implement because of the probable addition of spurious anticorrelations (Murphy *et al*., 2009), and the motion exclusion criteria, which we applied in order to diminish movement confounds (Baggio *et al*., 2015), could also contribute to the variation in reported results.

The SMA seed, however, was able to find significant differences between the patient groups and HCs. Both groups showed similar regional alterations in the precentral and postcentral gyri, precuneal, temporal, occipital and cerebellar regions. These FC alterations were more extensive in PDD than in DLB when compared with HCs, where such broader alterations may reflect the greater levels of Parkinsonism and motor dysfunction in PDD compared with DLB.

### No differences in FC between DLB and PDD

The 12 seeds assessed in this study were aimed at finding differences from regions related to the DMN, FPN and MN, but did not show any significant results. Although this outcome could be explained by the well‐documented similarities between DLB and PDD, we assessed regions based on reported clinical differences in attention/executive and memory functions, as well as level of Parkinsonism between both dementias. Reasons for the present findings could be again low sensitivity of the technique and the relatively low sample size. However, we were able to find differences between both patient groups and HCs for cortical seeds, suggesting that our analysis is sensitive when comparing patients against HCs, but not when comparing DLB and PDD, and this may be because of the overwhelming phenotypic overlap between both dementias.

## Conclusions

In the present study we analysed FC alterations in DLB and PDD and observed no significant functional differences between both dementia groups supporting the notion that at the functional level, PDD and DLB are broadly similar.

However, when both dementia groups are compared with HC, broader FC alterations were demonstrated in DLB in FPN‐related seeds, whilst broader FC alterations were apparent in the SMA seed in PDD. Our results therefore suggest that whilst both diseases are broadly similar, there are subtle underlying functional differences that may be driven by their different pathological trajectories.

## Conflict of interest

None declared.

Key point

- Dementia with Lewy bodies (DLB) and Parkinson's disease with dementia (PDD) are two dementias with overlapping symptoms and neuropathology.
- We assessed two cognitively matched PDD and DLB patient groups with mild to moderate impairment using resting state fMRI.
- Comparisons of both disease groups show differences in FC when compared with HC, which might be related to attention‐executive impairment and Parkinsonism in both diseases.
- However, when directly comparing DLB against the PDD group, we were not able to find significant differences in the resting state, suggesting that any functional difference between the two conditions is likely to be small.
